# Successful nutritional control of scratching and clinical signs associated with adverse food reaction: A randomized controlled COSCAD'18 adherent clinical trial in dogs in the United Kingdom

**DOI:** 10.1111/jvim.16192

**Published:** 2021-06-11

**Authors:** James L. Weemhoff, Jennifer M. MacLeay, John Brejda, Heidi Schiefelbein, Susan M. Wernimont, Kathy L. Gross

**Affiliations:** ^1^ Hill's Pet Nutrition, Inc Topeka, Kansas 66616 USA; ^2^ Alpha Statistics Lincoln Nebraska USA

**Keywords:** activity monitor, canine adverse food reactions, egg, novel protein, pruritus

## Abstract

**Background:**

Adverse reactions to food are a common dermatological condition in dogs, requiring nutritional intervention using novel or hydrolysate protein‐based foods.

**Objective:**

To evaluate a therapeutic food containing egg and phytonutrients in dogs with food allergies using an activity monitor and core outcome set for canine atopic dermatitis (COSCAD'18) in a controlled double‐masked, multicenter, prospective clinical trial.

**Animals:**

Adult dogs with a history of adverse food reaction as diagnosed by a food elimination trial were recruited from general practices.

**Methods:**

After a 21‐day baseline period, dogs were randomized to test or positive control (hydrolyzed protein) food for 21 days. Owner (pruritus visual analog score [PVAS], coat quality, food acceptance, and satisfaction) and veterinarian (canine atopic dermatitis lesion index [CADLI], physical examination) assessments were completed on days 0, 21, and 42. Dogs wore a collar‐mounted activity monitor to record sleep, scratching, and shaking behavior throughout the study. Statistical analysis included within‐group comparison to baseline and between‐group comparison at study end using a significance threshold of alpha = 0.05.

**Results:**

At the end of the treatment period, all results were similar between groups for CADLI, PVAS, owner satisfaction, activity, and questionnaire data. Scores for hair dullness, brittleness, amount of dandruff, feces quality, and food acceptance were positive and were not statistically different between groups.

**Conclusions and Clinical Importance:**

The therapeutic test food was well‐accepted and efficacious in managing signs of adverse reactions to food compared to baseline as well as compared to the positive control food.

**Abbreviations**
AICC: corrected Akaike information criterion
BFI: body fat index
BIC: Bayesian information criterion
CADESI: canine atopic dermatitis extent and severity index
CADLI: canine atopic dermatitis lesion index
CAFR: canine adverse food reaction
CBC: complete blood count
CI: confidence interval
COSCAD'18: 2018 core outcome set for canine atopic dermatitis
GI: gastrointestinal
OGATE: owner global assessment of treatment efficacy
PCF: positive control food
PCFB: period change from baseline
PVAS: pruritus visual analog score
SD: standard deviation
TTF: therapeutic test food

## 1 INTRODUCTION

Canine adverse food reaction (CAFR) accounts for about 10% of dogs presenting for pruritus or allergic skin disease^1^ and 15% of those may be allergic to chicken whereas only 4% seem to be allergic to egg.^2^ Clinical signs of CAFR typically are dermatological, gastrointestinal (GI), or both and affected dogs can be successfully managed using either alternate or hydrolyzed protein sources.^3^, ^4^

Controlled studies of CAFR are uncommonly reported in the literature and are not standardized. Lack of consistency in the design of interventional dermatology studies in dogs led to the proposal of the core outcome set for canine atopic dermatitis (COSCAD'18) which includes 3 endpoints: a veterinary assessment of skin lesions such as the canine atopic dermatitis lesion index (CADLI) or canine atopic dermatitis extent and severity index (CADESI), an owner‐assessment of scratching behavior such as the pruritus visual analog score (PVAS), and an owner global assessment of treatment efficacy (OGATE).^5^ We used COSCAD'18 criteria to evaluate 2 foods because the criteria could readily be applied to dogs with CAFR as well as dogs with atopic dermatitis. The major advantage of COSCAD'18 is that it allows for comparison of results between studies by standardizing outcomes. A drawback is that the measures used are subject to biased reporting by the veterinarian or a pet owner or both. Therefore, we included a wearable device to provide objective data. We previously described behavior algorithms for dogs based on continuous, high‐resolution accelerometer data collected by collar‐worn activity monitors.^6^ The algorithms are sensitive, specific, and >99% accurate in quantifying scratching and shaking in dogs based on validation against >500 video assessments of scratching and shaking in a population of >300 dogs.^6^ Activity monitors are capable of continuously and objectively quantifying behaviors in dogs 24 hours per day, 7 days per week, and have been used successfully in a nutrition intervention study.^7^

Our objective was to conduct a randomized controlled clinical trial using COSCAD'18 criteria and activity monitor data to evaluate the efficacy of a therapeutic food containing egg and sources of phytonutrients compared to a hydrolyzed protein control food in managing clinical and owner‐assessed pruritic signs in dogs with CAFR in a general practice setting.

## 2 MATERIALS AND METHODS

### 2.1 Ethics

This study was approved by the sponsor's Institutional Animal Care and Use Committee (CP824a, 11/17/2018) and was performed in accordance with the sponsor's animal welfare policy. Written, informed owner consent was obtained before the commencement of any study activities.

### 2.2 Study design

A controlled, double‐masked, multicenter, prospective clinical study was conducted and included monitoring of activity, CADLI,^8^ PVAS,^9^, ^10^ OGATE,^5^ and additional questionnaires. The study consisted of 2 periods: a baseline period (days 0‐21) and a treatment period (days 22‐42).

### 2.3 Patients

Recruitment occurred between September 2019 and March 2020 and included dogs ≥1 year of age if they were free of concurrent disease, had a history of GI signs (tenesmus, diarrhea, or soft feces) with or without dermatological signs (erythema, scratching) related to an adverse reaction to food diagnosed by resolution of signs after a feeding trial and were currently stable for these clinical signs as determined by the attending veterinarian; patients with known allergy to egg were excluded. Recruitment focused on dogs with cutaneous adverse reaction to food but those with concurrent GI manifestations were permitted provided the patient's GI signs were not consistent with inflammatory bowel disease (eg, undetermined weight loss, hypoproteinemia). Sample size calculations were made assuming a 40% change from baseline for the response variables. Monoclonal antibody‐based medications and intermittently‐dosed immunosuppressive agents were not allowed; other prior medications were allowed if owners were willing to keep the treatment regimen constant throughout the study. Pet owners were recruited by veterinarians from 11 general practices throughout the United Kingdom.

### 2.4 Study foods

Details of the macronutrient content and ingredient list of the study foods are provided as supplemental information (Tables S1 and S2); the positive control food (PCF) was a protein hydrolysate‐based food (Royal Canin Anallergenic, Lot #122027, Batch Codes 15/11/20, Purchased 06/2019) and the therapeutic test food (TTF) was a novel protein food with egg and rice as the major protein sources with additional sources of phytonutrients (Hill's Pet Nutrition Prescription Diet Derm Complete, Lot #122173, Produced 7/2019). Dogs were limited to their typical food during baseline and blocked based on CADLI scores (3 point increments). Patients were randomly assigned to either the TTF or PCF in a 1 : 1 allocation ratio using an electronic data capture system (Vision V9P3, Prelude Dynamics, Austin, Texas). Study foods were provided in color‐coded bags to mask food identity to all study participants. Dogs included in the analysis entered the study on a variety of foods, but all foods were novel or hydrolyzed protein foods or other limited ingredient or therapeutic foods. Supplements or treats were not allowed and dog owners were instructed to maintain the dog's feeding routine. The volume of food offered was based on the dog's current weight, caloric content of the study foods, and input from the veterinarian.

### 2.5 Activity monitor

All dogs enrolled in the study were provided with a collar‐mounted activity monitor which used triaxial accelerometer technology to generate a continuous record of the dog's motion throughout the baseline and treatment periods of the study. After collection, the data were processed using validated algorithms to quantify dermatologic related behaviors including scratching and shaking; sleeping and sleep quality (based on sleep disruptions) also were measured.^6^ Study days in which a minimum of 20 hours of data from the activity monitor was obtained were considered “qualifying days” and included in the statistical analysis. Behavior data were summarized as the total duration of each behavior in seconds per day whereas sleep quality was assessed on a scale of 0 (frequently interrupted) to 100 (uninterrupted).

### 2.6 Data collection

Study entry was considered day 0; questionnaire data from day 21 was considered baseline and compared to day 42 (end of study). Activity data were recorded on a continuous basis, the baseline period spanning days 0‐21, and test period days 22‐42. At each visit, veterinarians completed the CADLI and other questionnaires. For the CADLI, grades were assigned reflecting the degree of alopecia/lichenification/hyperpigmentation and erythema/excoriation/erosion for each of 5 body regions on a scale of 0 (none) to 5 (severe) yielding 2 subtotals that were added together for a maximum score of 50.^8^ Scores <8/50 were considered clinically normal.^5^ At the same time points, owners completed the PVAS using the scale of 0 (none) to 10 (severe).^9^, ^10^ Scores <3.6/10 were considered clinically normal.^5^ Owners were asked to score overall skin and fecal quality and answered a series of questions regarding their dog's quality of life based on a questionnaire developed and validated for dogs with atopic dermatitis.^11^ Quality of life responses consisted of a 5‐point Likert scale ranging from strongly agree to strongly disagree. Other questions were based on a score of 1‐100. Fecal quality was graded 1 (watery) to 5 (firm) using a scale that included both pictures and word descriptions. A successful food transition was defined as a transition to the study food without experiencing an adverse event or reporting poor palatability within 7 days of starting the study food, as reported by either the veterinarian or the owner.

### 2.7 Statistical methods

All data were analyzed by a biostatistician (J. Brejda) who was blinded to the identity of the test and control foods and using Statistical Analysis Software (SAS, version 9.4). Differences in mean animal age and weight among dogs assigned to PCF and TTF were analyzed using a 2‐sample *t* test. Distribution of body fat index (BFI) and sex between dogs enrolled into the PCF and TTF groups were analyzed using Fisher's exact test. Questionnaires that provided a numerical score ranging from 0‐100 were assumed to be continuous and normally distributed and were analyzed using a linear mixed model with diet, day, and diet × day as fixed effects in the model. The correlation between the repeated measures (day) was modeled by fitting an appropriate covariance structure selected using the corrected Akaike information criterion (AICC) and Bayesian information criterion (BIC) fit statistics. A simulation‐based adjustment was used to control for inflation of the Type I error rate resulting from multiple comparisons. Trends over days were tested using orthogonal polynomial contrasts for linear and quadratic trends. The pet quality of life questionnaire had nominal ordinal responses such as strongly agree, agree, neutral, disagree, or strongly disagree and was analyzed using the Cochran‐Mantel‐Haenszel test with modified ridit scores. Separate analyses were performed for each day the questionnaire was completed.

Values from wearable data for each activity during the 3‐week baseline period were averaged to produce a single mean value for each animal. Values from the treatment period were averaged at weekly intervals for each animal. The mean baseline values were subtracted from weekly treatment period values for each activity to calculate a period change from baseline (PCFB) value for each animal and week. The PCFB values were analyzed using a general linear mixed‐model with diet, week, and the diet × week interaction as fixed effects. To account for the correlation between repeated measurements made at weekly intervals, 6 common covariance structures were fit to the data, and the AICC fit statistic was used to select the best covariance structure for each activity. To control for multiplicity, a simulation‐based adjustment (ADJUST = SIMULATE) was used to control for inflation of the Type I error rate. To test for trends over time, orthogonal polynomial contrasts for linear, quadratic, and cubic effects were performed. Differences were considered significant when *P* ≤ .05.

## 3 RESULTS

### 3.1 Dogs

Patient signalment and characteristics are summarized and provided as supplemental information (Table S3). No significant differences were found between groups with respect to sex, weight, age, or breed. Thirty‐two dogs were screened, 29 were enrolled and 28 dogs completed the study and were included in the analysis (13 PCF and 15 TTF).

One dog (PCF) failed to complete the study due to deterioration of its dermatologic condition. Thirteen adverse events were reported for 8 dogs (4 PCF, 4 TTF) and were assessed by the attending investigator to be possibly or probably related to a food and ranged from mild to moderate and all resolved; 1 of these dogs ultimately was withdrawn from the study by its owner (1 PCF). The most common deviations reported were study visits conducted outside of the study design windows. Additional deviations included 14 instances in 9 dogs (4 PCF, 5 TTF) in which medications that may have had an effect on clinical signs were changed during the study period. These dogs still were included in the analysis because the timing or brevity or both of medication administration would not be expected to affect the response to the questions at subsequent visits. The details of these medications are provided in Table S4.

### 3.2 COSCAD outcomes

#### 3.2.1 Canine atopic dermatitis lesion index

**FIGURE 1:**
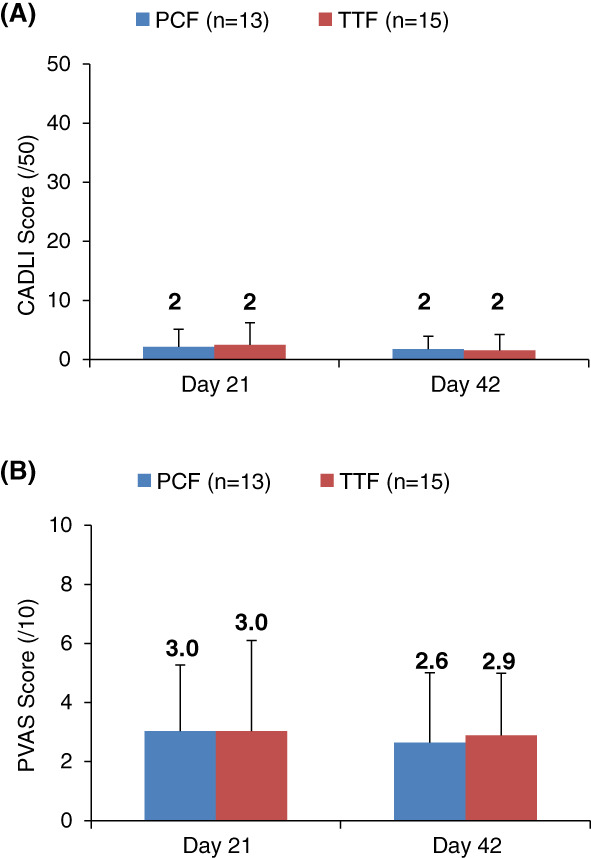
Comparison of canine atopic dermatitis lesion index (CADLI) (A) and pruritus visual analog (PVAS) (B) scores between therapeutic test food (TTF) and positive control food (PCF) after baseline and treatment periods. Data are expressed as mean ± SD. CADLI: PCF vs TTF: mean ± SD: Day 21 = 2 ± 3 vs 2 ± 4, Day 42 = 2 ± 2 vs 2 ± 3; *P* > .05. PVAS: PCF vs TTF: mean ± SD: Day 21 = 3.0 ± 2.2 vs 3.0 ± 3.1, Day 42 42 = 2.6 ± 2.4 vs 2.9 ± 2.1). No differences were significant between or within groups (*P* < .05)

No significant differences in CADLI scores were found between and within the PCF and TTF groups at end of baseline and end of study, and results are shown in Figure 1A. Most dogs entered and completed the study with a CADLI <8 (24/28; 86%), indicating a good level of control.^5^ Dogs that entered the study with a CADLI ≥8 (2 PCF, 2 TTF), ended the study with scores ≤8 except 1 TTF dog (start, 9/50; end, 10/50).

#### 3.2.2 Pruritus visual analog score

No significant differences in PVAS scores were found between or within groups at any of the visits (Figure 1B). Most dogs (19/32; 59%) entered and completed the study with PVAS scores <3.6/10. Nine dogs began with a score >3.6 (4 PCF, 5 TTF) and of these, all finished the study with improved scores except 1 TTF dog (start, 4.1; end, 6.7).

#### 3.2.3 Owner assessments

Outcomes are reported in Tables 1 and 2. Significantly less disruption of the dog's skin condition on the family's sleep and less interference in the owner's enjoyment of snuggling with their dog were found in the TTF group when compared to baseline. No other statistical differences (*P* > .05) were found between or within groups of owner‐reported dermatological or quality of life metrics.

**TABLE 1:** Owner reported outcomes for treatment efficacy

Question	Scale	Description	Baseline		End of study	
			PCF	TTF	PCF	TTF
How dull is your dog's hair coat?	0‐100	Very shiny‐very dull	13.0 ± 10.4	32.0 ± 18.9	9.8 ± 8.8	30.8 ± 17.6
How coarse or brittle is your dog's hair?	0‐100	Very soft‐very coarse/brittle	20.8 ± 19.7	40.0 ± 27.5	11.4 ± 17.4	31.3 ± 23.2
How much dandruff is your dog currently displaying?	0‐100	None‐extremely flaky/scaly	4.5 ± 11.1	10.1 ± 19.4	2.6 ± 4.8	8.9 ± 19.6
How disruptive is your dog's skin condition currently to you or your family?	0‐100	Not disruptive‐extremely disruptive	10.3 ± 12.9	24.1 ± 33.3	9.9 ± 19.4	18.6 ± 22.3
How disruptive is your dog's skin condition currently to you or a member of your family's sleep?	0‐100	Not disruptive‐extremely disruptive	7.7 ± 21.6	21.7 ± 34.7	9.3 ± 21.7	8.1 ± 19.2*
How much does your dog's skin condition interfere with your enjoyment of snuggling with your dog?	0‐100	Not disruptive‐extremely disruptive	3.5 ± 9.4	10.5 ± 2.3	1.5 ± 3.7	2.3 ± 4.9*
Feces score	0‐5	Watery‐firm	4.7 ± 0.5	4.5 ± 0.6	4.9 ± 0.3	4.6 ± 0.5

*Note*: Values are represented as mean ± SD.

Abbreviations: PCF, positive control food; TTF, therapeutic test food.

* *P* < .05 vs baseline.

**TABLE 2:** Quality of life assessment

Question	Change	PCF	TTF
My dog interrupts sleeping in order to scratch, lick, bite, or chew itself.	Improvement	3	5
	No change	6	5
	Worse	2	2
My dog is happy.	Improvement	1	1
	No change	10	7
	Worse	0	4
My dog is playful and active.	Improvement	1	1
	No change	10	7
	Worse	0	2
My dog is tired because of his disease.	Improvement	4	2
	No change	5	6
	Worse	2	4
My dog sleeps well.	Improvement	1	2
	No change	8	7
	Worse	2	3
The skin disease has changed my dog's behavior for the worse.	Improvement	4	3
	No change	5	7
	Worse	2	2
The treatment itself (shampoo, pills, etc.) is a major burden to my dog.	Improvement	4	2
	No change	6	5
	Worse	1	5

*Note*: Number of dog owners reporting improved, same (no change), or worse scores for quality of life related questions at study start vs study end. No proportions were statistically different for control food vs therapeutic test food.

Abbreviations: PCF, positive control food; TTF, therapeutic test food.

### 3.3 Activity data

Dogs tolerated the use of the activity sensors and owners did not report any issues with their pets wearing the device. Activity data could not be obtained from 7 dogs (3 PCF, 4 TTF) for technical reasons. Compliance was excellent: the average number of qualifying days (days with >20 hours of wearable data) was 78% for the baseline period and 89% for the treatment period. Comparing change from baseline between treatment groups (PCF vs TTF), no significant differences were found in any of the evaluated behaviors: scratching, shaking, sleep duration, and sleep quality index (Table 3).

**TABLE 3:** Weekly animal activity data for dogs randomized to the therapeutic test food and positive control food groups presented as change from baseline

Behavior	Week	PCF		TTF	
		Mean	95% CI	Mean	95% CI
Scratching (s/d)	1	−2.95 ± 35.4	−76.9 to 71.0	−23.6 ± 33.7	−94.1 to 46.9
	2	−22.3 ± 36.4	−98.6 to 54.0	−13.9 ± 34.7	−86.6 to 58.9
	3	−25.5 ± 56.2	−144.9 to 93.9	−10.7 ± 55.1	−127.1 to 105.7
Shaking (s/d)	1	2.16 ± 3.12	−4.38 to 8.70	0.68 ± 2.98	−5.56 to 6.91
	2	−3.38 ± 4.33	−12.48 to 5.72	−4.5 ± 4.12	−13.18 to 4.18
	3	1.50 ± 5.71	−10.54 to 13.53	−6.58 ± 5.55	−18.23 to 5.07
Sleeping (h/d)	1	0.05 ± 0.17	−0.3 to 0.4	0.09 ± 0.16	−0.24 to 0.43
	2	−0.06 ± 0.26	−0.61 to 0.49	0.23 ± 0.25	−0.29 to 0.75
	3	−0.10 ± 0.23	−0.57 to 0.38	−0.05 ± 0.24	−0.56 to 0.45
Sleep quality index	1	0.79 ± 1.01	−1.33 to 2.91	1.81 ± 0.97	−0.21 to 3.83
	2	−0.26 ± 1.91	−4.25 to 3.73	1.48 ± 1.82	−2.33 to 5.28
	3	0.67 ± 2.28	−4.13 to 5.48	2.78 ± 2.23	−1.90 to 7.47

*Note*: No changes were significantly different between or within groups. Data are represented as mean ± SD and confidence interval (CI).

Abbreviations: PCF, positive control food; TTF, therapeutic test food.

#### 3.3.1 Other metrics

Veterinarians were asked a series of questions to evaluate the dog's overall skin and hair coat health. At the end of the study, skin healing was significantly better in dogs consuming the TTF (there was no significant change over time for the PCF group, Table 4). Successful transition to the study foods was higher for the TTF (15/15, 100%) than the PCF (13/14, 93%) but was not significantly different. Owner responses to food‐related questions are presented in Table 5. The aroma strength of the TTF was significantly lower when compared to the PCF (end of study) and significantly better than their previous food (baseline). All other measures were not different between or within groups.

**TABLE 4:** Veterinary reported outcomes for skin and coat health

Question	Scale	Description	Baseline		End of Study	
			PCF	TTF	PCF	TTF
The amount of shedding.	0‐100	None‐severe	17.4 ± 25.5	11.3 ± 16.3	11.0 ± 13.5	13.4 ± 21.4
The overall skin quality.	0‐100	Excellent‐poor	12.9 ± 16.1	15.3 ± 13.9	8.9 ± 9.9	20.9 ± 29.9
The overall coat quality.	0‐100	Excellent‐poor	10.3 ± 11.6	13.9 ± 13.9	9.6 ± 9.9	14.0 ± 15.9
The overall clinical signs associated with dermatitis.	0‐100	None‐severe	12.5 ± 16.3	16.3 ± 22.3	12.3 ± 14.8	15.4 ± 19.1
How the overall clinical signs associated with dermatitis in this dog have changed since the previous exam?	0‐100	Significant improvement‐significant deterioration	46.3 ± 9.7	47.0 ± 13.8	43.1 ± 17.3	35.8 ± 23.0
How has skin healing progressed since the previous exam?	0‐100	Significant improvement‐significant deterioration	45.5 ± 8.6	50.0 ± 0.7	39.3 ± 16.9	33.6 ± 20.3*

*Note*: No scores were significantly different within or between groups. Values are represented as mean ± SD. Scores were 0‐100 with the lower score reflecting more ideal status.

Abbreviations: PCF, positive control food; TTF, therapeutic test food.

* *P* < .05 vs baseline.

**TABLE 5:** Owner reported outcomes for food assessment and performance

Question	Scale	Description	Baseline		End of study	
			PCF	TTF	PCF	TTF
The strength of the aroma of the food you are feeding your dog.	0‐100	No aroma‐extremely strong aroma	47.1 ± 30.4	41.9 ± 27.5	72.5 ± 22.5	45.5 ± 26.1**
The pleasantness of the aroma of the food you are feeding your dog.	0‐100	Not pleasant‐extremely pleasant	62.8 ± 18.3	45.2 ± 21.8	62.2 ± 29.2	69.0 ± 15.5*
Owner—How would you rate your satisfaction with the current food?	0‐100	Most satisfied‐least satisfied	…		23.4 ± 26.5	27.1 ± 26.7
Owner—Do you prefer this food over the dog's previous food?	Yes/No	…	…		70%	50%
Owner—Would you recommend this food to a friend?	Yes/No	…	…		85%	91%
Veterinarian—How would you rate your satisfaction with the current food?	0‐100	Most satisfied‐least satisfied	…		23.4 ± 26.5	27.1 ± 26.7
Veterinarian—Would you recommend this food to a colleague?	Yes/No	…	…		91%	75%

*Note*: Values are represented as mean ± SD where appropriate. Scores were 0‐100. Several questions were only assessed at study end.

Abbreviations: PCF, positive control food; TTF, therapeutic test food.

* *P* < .05 vs baseline.

** *P* < .05 vs positive control.

## 4 DISCUSSION

We documented similar control of signs associated with CAFR between foods in owner‐dog pairs managed in general practices in the United Kingdom using a core outcome set of measurements developed and endorsed by veterinary dermatologists^5^ in conjunction with an activity monitor. Similarly, we chose the CADLI, PVAS, and owner evaluation results as primary outcome measures but also used activity data and veterinary outcomes to supplement these findings. A shortcoming of our study was that the population of dogs was not described based on the specific source of their food allergy. A practical approach for general practitioners is to recommend avoidance of common food allergens by prescribing foods with novel or hydrolyzed protein or both, and the results of our study support that approach. To our knowledge, a comparison showing similar outcomes between a hydrolyzed and a novel protein food in a population of dogs with CAFR managed in clinical practice has not been published previously.

The TTF incorporated ingredients to avoid as well as modulate the immune response. For the test food, egg was selected as a primary protein source because documented cases of allergies to egg represent approximately 4% of all food allergies in dogs, lower than other protein sources such as chicken or soy.^12^, ^13^, ^14^ Several components of egg have been shown to have immunomodulating.^15^ We previously have reported that a food with polyphenol containing phytonutrients modulates clinical signs in dogs with environmental allergies.^16^, ^17^ Polyphenols have been shown to modulate the immune system directly by inhibiting degranulation of mast cells, as well as cytokine and immunoglobulin production and indirectly by binding antigens, thus rendering them less recognizable by the immune system.^18^, ^19^ Clinically, once pruritus is controlled, skin healing can commence and is supported by dietary antioxidants, vitamins, fatty acids, and minerals.^20^, ^21^, ^22^, ^23^

To our knowledge, ours is the first study using COSCAD'18 criteria to evaluate food in managing CAFR. The COSCAD'18 was intended to be used in the evaluation of pharmaceuticals in dogs with environmental allergies but its use for food allergy has worked well. A benefit of the COSCAD'18 is that it demands a broader assessment of the clinical condition than a study design using only veterinary or owner input. Furthermore, our study showed that COSCAD'18 could be employed in study designs examining the maintenance of clinical condition.

We interpreted the CADLI and PVAS with reference to values considered normal because some level of scratching behavior can be habitual. The upper threshold between what would be considered normal and what would be clinically relevant is a CADLI score of 8 and a PVAS score of 3.6.^5^ To be eligible, dogs were considered controlled with respect to dermatological signs, but minimum criteria were not set nor were dogs blocked based on score. Different scores were observed among participants at study entry but were not different between groups. The range was likely because of the fact that controlled is a subjective assessment. Dogs entering the study with higher scores improved and improvements were similar between groups.

To our knowledge, ours is the first study to combine COSCAD'18 criteria with activity data in a nutrition intervention study. Activity monitors provided continuous and objective quantification of each dog's pruritic behavior. One limitation is that minimum technological requirements must be met, which may prohibit its use by certain owners and 1 participant was lost for this reason. Wearable data supported that both foods maintained control of clinical signs including scratching, shaking, sleep duration, and sleep quality. Future studies could examine the correlation between individual COSCAD'18 criteria and measures of behavior in larger groups of dogs with active clinical signs.

Multiple owner‐assessed outcomes were evaluated, including hair coat dullness, brittleness, and the amount of dandruff, as well as endpoints reflecting companionship. Owners of dogs in the TTF group reported less disruption and greater improvements in desire to snuggle and veterinarians reported significant healing compared to baseline. Other measures were similar between and within groups. Dogs began the study with normal fecal quality which did not change, and foods were similarly accepted in both groups. When asked specifically about their opinion of the foods, veterinarians and owners reported high satisfaction scores (>50/100) and willingness to recommend foods to a friend or colleague (>50%).

One limitation of our study is that the elimination trial was performed previously and not as part of the trial. As such, we were not able to set the conditions of the food elimination trial. This was because a prospective trial to recruit newly diagnosed cases of CAFR would take a long period of time. We controlled for this issue by recruiting animals where the veterinarian, and not the owner, attested to the presence of the food allergy and history of a food elimination trial. A second limitation is that enrolled dogs could have been concurrently treated with medications and dogs with a history of possible environmental allergies were not excluded. We tried to control this issue by stipulating that owners must hold treatment regimens constant. Regardless, 3 dogs (1 PCF, 2 TTF) received either corticosteroids (PCF) or oclacitinib (2 TTF) during the baseline (PCF) or treatment (TTF) phase. These dogs were not removed from the analysis because after an evaluation of the medical records, it was determined that the medication was not given because of the food's efficacy. The dog that received corticosteroids developed an aural hematoma during the baseline period while consuming its original food. The 2 dogs that received oclacitinib received this medication for increased scratching 13 (CADLI 4 at time of prescription) and 17 (CADLI score of 16 at time of prescription) days after the initiation of the study diet. A primary reason to include them in the analysis was a review of the medical records, which could not rule out environmental allergy and the timing of the visit. According to a review of 234 dogs, 80% of dogs have a flare within 7 days of a food challenge and >90% by day 14. Continued feeding of either food in these 3 cases was not associated with early study withdrawal or with worsening signs at study end.^24^ A final potential limitation to our study is the possibility of Type II error. To minimize this possibility, sample size calculations were made assuming a 40% change from baseline for the response variables. This is the magnitude of response anticipated as a result of feeding the therapeutic food. The anticipated response was observed over time, which from a clinical standpoint was the desired outcome because it shows the therapeutic food was successful. However, it is possible that the magnitude of the difference between the 2 foods was not as large as the magnitude of change over time. As a result, the sample size needed to show a significant change over time was insufficient to identify significant differences between the 2 foods, resulting in Type II error.

## 5 CONCLUSION

In summary, we successfully used COSCAD'18 criteria and activity monitors to provide evidence for similar management of clinical signs associated with CAFR by 2 foods: a commercially available hydrolyzed protein‐based food and a therapeutic food with egg, rice, and sources of polyphenols.

## CONFLICT OF INTEREST DECLARATION

J. Brejda provided contracted services to Hill's Pet Nutrition, Inc, and all other authors are employees of Hill's Pet Nutrition, Inc. Preliminary results from this study were presented as 2 abstracts of The 9th World Congress of Veterinary Dermatology. Evaluation of clinical signs related to adverse food reaction (AFR) in dogs using a new behavior‐recognition approach and Successful management of clinical signs related to adverse food reaction in dogs using a food with a new approach. *Veterinary Dermatology* 2020: 31:S1:103‐104.

## OFF‐LABEL ANTIMICROBIAL DECLARATION

Authors declare no off‐label use of antimicrobials.

## INSTITUTIONAL ANIMAL CARE AND USE COMMITTEE (IACUC) OR OTHER APPROVAL DECLARATION

This study was approved by Hill's Pet Nutrition IACUC (CP824a, 11/17/2018) and was performed in accordance with the Hill's Pet Nutrition animal welfare policy. Written, informed owner consent was obtained prior to commencement of any study activities.

## HUMAN ETHICS APPROVAL DECLARATION

Authors declare human ethics approval was not needed for this study.

## Supporting information


**Table S1** Macronutrient content of study foods reported on a dry matter basis.
**Table S2**. Ingredient lists of both therapeutic and positive control study foods.
**Table S3**. Patient demographics for adult dogs diagnosed with adverse reaction to food.
**Table S4**. Details of concurrent medications that were changed during the study and which may have affected clinical sign.
**Figure S1**. CONSORT flow diagram of patient screening, exclusion, and withdrawals.
**Figure S2**. Comparison of CADLI (A) and PVAS (B) scores between therapeutic test food (TTF) and positive control food (PCF) at Day 0, Day 21 (baseline), and Day 42 (treatment).
