# Supplementary material for: Successful nutritional control of scratching and clinical signs associated with adverse food reaction: A randomized controlled COSCAD'18 adherent clinical trial in dogs in the United Kingdom
Source: J Vet Intern Med. 2021 Jun 11;35(4):1893–901. doi: 10.1111/jvim.16192 (PMC8295676; doi:10.1111/jvim.16192)

SUPPLEMENTAL TABLE

Table S1: *Macronutrient content of study foods reported on a dry matter basis. PCF= positive control food, TTF= therapeutic test food*

	**PCF**	**TTF**
**Caloric Content (kcal/kg)**	4945	5084
**Crude Protein (%)**	20	18
**Crude Fat (%)**	17	16
**Crude Fiber (%)**	1.9	1.3
**Ash (%)**	7.0	5.2

Table S2: *Ingredient lists of both therapeutic and positive control study foods.*

Study Food	Ingredient List
**Therapeutic Test Food (TTF)**	Cereals, oils and fats (including soya oil, copra oil, fish oil), eggs and egg derivatives, meat and animal derivatives, vegetable protein extracts, seeds, minerals, derivatives of vegetable origin, vegetables, fruit, vitamin A, vitamin D3, vitamin E, vitamin C, beta-carotene, iron, iodine, copper, manganese, zinc, selenium, natural antioxidants.
**Positive Control Food (PCF)**	Maize starch, feather hydrolysate with low molecular weight (source of L-amino acids and oligopeptides), copra oil, soya oil, minerals, vegetable fibres, chicory pulp, fructo-oligo-saccharides, fish oil, mono- and diglycerides of palmitic and stearic acids esterified with citric acid, animal fat, marigold extract (source of lutein), vitamin A, vitamin D3, iron, iodine, copper, manganese, zinc, selenium, clinoptilolite of sedimentary origin, preservatives, antioxidants.

Table S3: *Patient demographics for adult dogs diagnosed with adverse reaction to food. Data are represented as absolute counts or as mean ± SD. Number of intact animals: † =1, ‡ = 2, § = 3, ¶ = 4. PCF= positive control food, TTF= therapeutic test food*

	**Completed**	**PCF**	**TTF**
**Total**	28	13	15
**Age (yr)**	5.5 ± 3.5	3.9 ± 2.6	6.9 ± 3.7
**Weight (kgs)**	20.2 ± 13.1	20.0 ± 12.7	20.5 ± 14.0
**Gender**			
**Male**	17 (61%)¶	8 (62%)‡	9 (60%)‡
**Female**	11 (39%)§	5 (38%)†	6 (40%)‡
**Breed**			
**Purebred**	19 (68%)	10 (77%)	9 (60%)
**Mixed**	6 (21%)	2 (15%)	4 (27%)
**Not Reported**	3 (11%)	1 (8%)	2 (13%)

T**able S4**: *Details of concurrent medications that were changed during the study and which may have affected clinical signs.*

Group	Patient	Enrollment	Day 21	Day 42	Medication Name	Medication Start	Medication Finish
Control	1	9/27/19	10/18/19	11/8/19	Chrlorphenamine	10/14/19	10/15/19
	2	10/22/19	11/12/19	12/3/19	Chlorphenamine	11/25/19	NA
	3	2/14/20	3/27/20	4/18/20	Florfenicol, terbinafine, betamethasone acetate otic solution	2/14/20	NA
	4	12/9/20	1/2/20	1/23/20	Miconazole, polymyxin B, prednisolone otic solution	8/1/17	12/23/19
	4	12/9/20	1/2/20	1/23/20	hydrocortisone, miconazole, gentamicin otic suspension	12/23/19	1/1/20
	4	12/9/20	1/2/20	1/23/20	Dexamethasone	12/23/19	12/23/19
	4	12/9/20	1/2/20	1/23/20	Depomedrone	12/24/19	1/14/20
Test	1	9/19/19	10/10/19	10/31/19	Chloramphenicol	10/18/19	10/22/19
	2	10/24/19	11/11/19	12/10/19	Chlorphenamine	11/28/19	11/29/19
	2	10/24/19	11/11/19	12/10/19	Oclacitinib	11/28/19	NA
	3	1/24/20	2/28/20	3/20/20	Oclacitinib	3/13/20	NA
	4	10/7/19	10/24/19	11/18/20	Amoxicillin, clavulanic acid	10/15/19	10/29/19
	5	1/28/20	2/18/20	3/10/20	Prednisolone	12/17/19	1/30/20
	5	1/28/20	2/18/20	3/10/20	Florfenicol, terbinafine, betamethasone acetate otic solution	2/7/20	2/27/20

Figure S1: *CONSORT Flow Diagram of patient screening, exclusion, and withdrawals.*

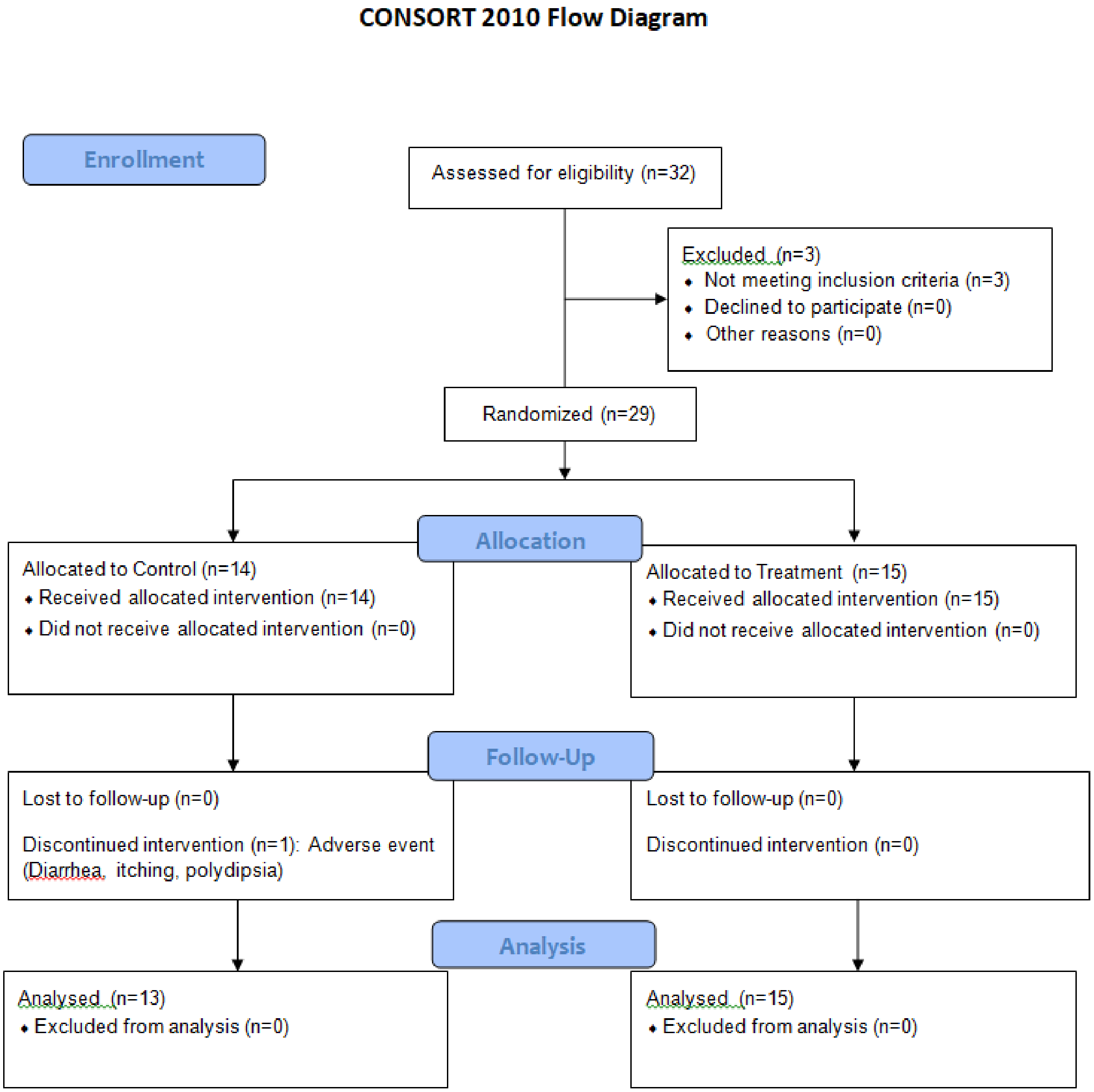

Figure S2: *Comparison of CADLI (A) and PVAS (B) scores between therapeutic test food (TTF) and positive control food (PCF) at Day 0, Day 21 (Baseline), and Day 42 (Treatment). Data are expressed as mean ± SD. CADLI: PCF vs. TTF: mean ± SD: Day 0 = 3 ± 3 vs. 2 ± 3, Day 21 = 2 ± 3 vs. 2 ± 4, Day 42 = 2 ± 2 vs. 2 ± 3; p>.05. PVAS: PCF vs. TTF: mean ± SD: Day 0 = 2.6 ± 2.4 vs. 4.2 ± 3.0, Day 21 = 3.0 ± 2.2 vs. 3.0 ± 3.1, and Day 42 = 2.6 ± 2.4 vs. 2.9±2.1; p>.05).*

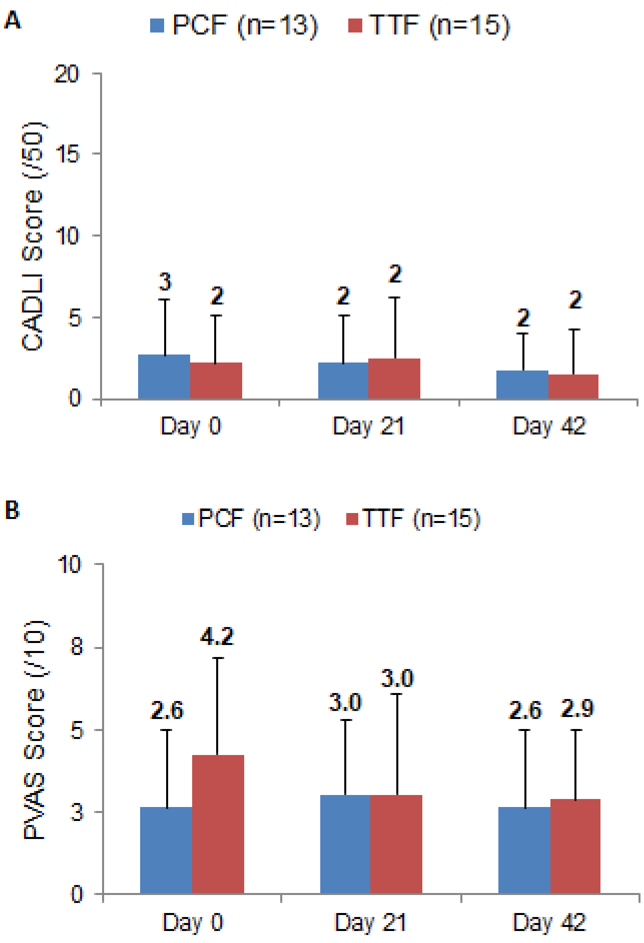

Supplement: Supplementary file 1 — Table S1 Macronutrient content of study foods reported on a dry matter basis. Table S2. Ingredient lists of both therapeutic and positive control study foods. Table S3. Patient demographics for adult dogs diagnosed with adverse reaction to food. Table S4. Details of concurrent medications that were changed during the study and which may have affected clinical sign. Figure S1. CONSORT flow diagram of patient screening, exclusion, and withdrawals. Figure S2. Comparison of CADLI (A) and PVAS (B) scores between therapeutic test food (TTF) and positive control food (PCF) at Day 0, Day 21 (baseline), and Day 42 (treatment). [file JVIM-35-1893-s001.pdf]
